# Cortical neuroanatomical changes related to specific language impairments in primary progressive aphasia

**DOI:** 10.3389/fnagi.2022.878758

**Published:** 2022-08-25

**Authors:** Sung Hoon Kang, Yu Hyun Park, Jiho Shin, Hang-Rai Kim, Jihwan Yun, Hyemin Jang, Hee Jin Kim, Seong-Beom Koh, Duk L. Na, Mee Kyung Suh, Sang Won Seo

**Affiliations:** ^1^Department of Neurology, Samsung Medical Center, Sungkyunkwan University School of Medicine, Seoul, South Korea; ^2^Department of Neurology, Korea University Guro Hospital, Korea University College of Medicine, Seoul, South Korea; ^3^Department of Health Sciences and Technology, SAIHST, Sungkyunkwan University, Seoul, South Korea; ^4^Department of Neurology, Dongguk University Ilsan Hospital, Dongguk University College of Medicine, Goyang, South Korea; ^5^Department of Digital Health, SAIHST, Sungkyunkwan University, Seoul, South Korea; ^6^Alzheimer’s Disease Convergence Research Center, Samsung Medical Center, Seoul, South Korea; ^7^Department of Intelligent Precision Healthcare Convergence, Sungkyunkwan University, Suwon, South Korea

**Keywords:** neural substrate, language function test, primary progressive aphasia, object naming, generative naming, comprehension, cortical atrophy

## Abstract

**Objective:**

Language function test-specific neural substrates in Korean patients with primary progressive aphasia (PPA) might differ from those in other causes of dementia and English-speaking PPA patients. We investigated the correlation between language performance tests and cortical thickness to determine neural substrates in Korean patients with PPA.

**Materials and methods:**

Ninety-six patients with PPA were recruited from the memory clinic. To acquire neural substrates, we performed linear regression using the scores of each language test as a predictor, cortical thickness as an outcome and age, sex, years of education, and intracranial volume as confounders.

**Results:**

Poor performance in each language function test was associated with lower cortical thickness in specific cortical regions: (1) object naming and the bilateral anterior to mid-portion of the lateral temporal and basal temporal regions; (2) semantic generative naming and the bilateral anterior to mid-portion of the lateral temporal and basal temporal regions; (3) phonemic generative naming and the left prefrontal and inferior parietal regions; and (4) comprehension and the left posterior portion of the superior and middle temporal regions. In particular, the neural substrates of the semantic generative naming test in PPA patients, left anterior to mid-portion of the lateral and basal temporal regions, quite differed from those in patients with other causes of dementia.

**Conclusion:**

Our findings provide a better understanding of the different pathomechanisms for language impairments among PPA patients from those with other causes of dementia.

## Introduction

Primary progressive aphasia (PPA) is a clinical syndrome characterized by focal neurodegeneration in the language-dominant cerebral hemisphere that leads to language impairment in the absence of other salient cognitive impairments. There are three PPA subtypes: non-fluent/agrammatic variant PPA (nfvPPA), semantic variant PPA (svPPA), and logopenic variant PPA (lvPPA), which are based on the nature of language impairment (Gorno-Tempini et al., 2011). Specifically, nfvPPA shows an abnormality of syntax/grammar or effortful/halting speech with apraxia of speech, svPPA has poor performance in single-word comprehension and confrontational naming, and lvPPA has word-finding hesitation and impaired repetition of sentences and phrases (Gorno-Tempini et al., 2011). During the course of the PPA diagnosis, patients undergo a series of language assessments using normalized language evaluation tools (ex. Western Aphasia Battery [WAB], Boston Naming Test [BNT]) and supporting tasks/tests to determine whether the patient has disrupted grammatical abilities, semantics, phonological processing, or other language-related processes.

The development of neuroimaging methods has enabled the detection of subtle cortical atrophy in neurodegenerative diseases. Specifically, three PPA subtypes showed distinct cortical atrophy patterns that correlated with language impairment patterns: left prefrontal atrophy in nfvPPA, left anterior temporal atrophy in svPPA, and left superior temporal and inferior parietal atrophy in lvPPA (Sapolsky et al., 2010; Gorno-Tempini et al., 2011). Especially, given that cortical atrophy precedes the onset of language impairment in patients with PPA (Rogalski and Mesulam, 2009), the language impairment-cortical atrophy correlation, called neural substrates of language impairment, is important in clinical practice, not only to understand a patient’s current language impairment but also to predict the progression of symptoms. Previous studies based on patients with PPA showed that performances in language fluency, comprehension, repetition, and object naming tests were mainly associated with cortical atrophy in specific regions (Rogalski et al., 2011a; Mesulam et al., 2014, 2019; Forkel et al., 2020).

However, the neural substrates of each language component differed slightly among the research groups (Amici et al., 2007; Rogalski et al., 2011a). The differences may have come from the different tasks the researchers used to detect the associated brain areas of a certain language function. Furthermore, there is a paucity of information about the atrophy patterns of the three PPA subtypes in the Korean population, although growing evidence reveals distinct cortical atrophy patterns based on PPA subtypes in the Western population. Given that the Korean language is quite different from English in, for example, grammar and generative syntax, the neural substrates of language tests may show different patterns between the Korean and English-speaking populations. Regarding grammar and generative syntax, the main differences between the two languages are word order, usage of postpositions/particles, and verb conjugations. Thus, these differences may reflect different neural substrates of fluency and atrophy patterns of nfvPPA in the Korean, compared to those in the English-speaking populations. Hence, it is necessary to reduce the knowledge gaps in the current understanding of cortical atrophy patterns of PPA subtypes and neural substrates of language impairments in different racial/ethnic populations, such as the Korean population. Previously, our group revealed the neural substrates of language impairment in Korean Alzheimer’s continuum (Albert et al., 2011; McKhann et al., 2011; Sperling et al., 2011) including preclinical Alzheimer’s disease (AD), mild cognitive impairment due to AD and AD dementia (Ahn et al., 2011; Kang et al., 2019) and subcortical vascular cognitive impairment (SVCI) (Kang et al., 2021). However, given that neural substrates are affected by cortical atrophy patterns in specific diseases, it might be noteworthy to examine the neural substrates of language tests in PPA and find a pattern which differs from previously reported results of patients with other causes of dementia.

Therefore, our study aimed to investigate language function test-specific neural substrates in a relatively large sample of Korean patients with PPA. First, we identified distinct cortical atrophy patterns in the three Korean PPA subtypes. Second, we explored the correlation between language performance across six domains of language, namely, language fluency, comprehension, sentence repetition, object naming, semantic generative naming, and phonemic generative naming, and cortical thickness in Korean patients with PPA.

## Materials and methods

### Study participants

We recruited 109 patients with PPA (35 patients with svPPA, 51 patients with nfvPPA, and 23 patients with lvPPA) from the dementia clinic in the Department of Neurology at Samsung Medical Center in Seoul, Korea, between October 2014 and December 2019. All patients met the core criteria for PPA (Gorno-Tempini et al., 2011) as follows: (1) language impairment being the earliest and most prominent clinical feature and (2) the principal cause of impaired activities of daily living, at initial phases of the disease. Based on the PPA consensus criteria (Gorno-Tempini et al., 2011), we classified the patients as having nfvPPA, svPPA, and lvPPA.

All participants underwent comprehensive dementia evaluation including a clinical interview, neurological examination, standardized neuropsychological test, standardized language function test, blood tests, and high-resolution T1-weighted magnetic resonance imaging (MRI) scans. The time interval between the language function tests and MRI scans was less than 1 year.

All participants were determined for handedness using a questionnaire “measurement of handedness in Koreans” (Kang, 1994), which included five questions in such as “Which hand do you use most when…?” There were five questions concerning writing, using chopsticks, throwing a ball, using a knife, and using scissors. Further, to exclude the forced right-handedness linked with cultural aspects, we defined individuals who wrote and/or used chopsticks with their right hand and did the rest of the items with their left hand as left-handedness.

We excluded patients with secondary causes of cognitive deficits confirmed with laboratory tests, including vitamin B12 test, syphilis serology, and thyroid/renal/hepatic function tests, and those with structural lesions on conventional brain MRI, such as territorial infarction, intracranial hemorrhage, brain tumor, hydrocephalus, or severe white matter hyperintensities (D3P3), according to the modified Fazekas ischemic scale (Noh et al., 2014). Patients clinically diagnosed with other types of degenerative diseases, such as AD, progressive supranuclear palsy, cortico-basal syndrome, behavioral variant frontotemporal dementia, or Lewy body/Parkinson’s disease dementia, were also excluded at baseline evaluation.

We also recruited 308 participants with normal controls (NC) as previously described (Kang et al., 2021), All participants with NC met the following criteria: (1) no medical history, which is likely to affect cognitive function based on Christensen’s health screening criteria (Christensen et al., 1991); (2) no objective cognitive impairment from comprehensive neuropsychological test battery on any cognitive domains (≥−1.0 SD of age- and education-matched norms on any cognitive tests); (3) independence in activities of daily living; (4) neither structural lesions nor severe white matter hyperintesity on brain MRI; and (5) negative amyloid deposition on positron emission tomography, by using visual assessment.

This study was approved by the institutional review board of the Samsung Medical Center. Written informed consent was obtained from all the patients or their caregivers.

### Language assessment

A total of 109 patients with PPA underwent the Korean version of the Western Aphasia Battery (K-WAB), a standardized language function test (Kim and Na, 2004). All patients were evaluated by certified linguistic pathologists, and the scoring system of most tests in the K-WAB remained the same as that of the original Western Aphasia Battery. However, some aspects of the rating scale have been changed, as previously described (Lee et al., 2020). Among the many language function tests evaluated in the K-WAB, we used tests that provided numeric scores and represented the six domains of language function: language fluency, comprehension, sentence repetition, object naming, semantic generative naming, and phonemic generative naming. For language fluency, spontaneous speech was scored using the 10-point fluency scale and 10 points for information content. The comprehension task (200-point) consisted of yes/no questions (60-point), auditory word recognition (60-point), and sequential command (80-point). Yes/no question and sequential commands tasks include a few syntactically-complex sentences. The repetition task consisted of single syllable word to sentences of six groupings in length with combinations of words and word + postpositions (10-point). For object naming (60-point), we used the 20 objects provided in the K-WAB battery. Object naming task is performed by asking the subjects/patients to name the objects shown to them, thus the task does not require overt responses of object knowledge. However, when patients are unable to respond or make errors spontaneously, after the semantic and phonemic cues are given, respectively, and the patient’s responses all marked, the patients are asked to describe what the object is for or how it is used, either verbally or through gestures. This was to check to see whether the patient’s object knowledge functions were preserved or not. For semantic generative naming, animal category was used, and phonemic generative naming consisted of Korean letters categories. Furthermore, we calculated aphasia quotient (AQ) to assess the overall severity of language impairment as a following formula: scores for information content and fluency in the subtest of spontaneous speech and the converted scores of comprehension, repetition and naming tasks all of which are 10 points, respectively, are added and then multiplied by 2 to make 100.

### Acquisition of three-dimensional magnetic resonance images

We acquired three-dimensional T1 Turbo Field Echo MRI scans of 109 patients with PPA using a 3.0 T MRI scanner (Philips 3.0T Achieva) with the following imaging parameters: sagittal slice thickness, 1.0 mm with 50% overlap; no gap; repetition time of 9.9 ms; echo time of 4.6 ms; flip angle of 8°; and matrix size of 240 × 240 pixels reconstructed to 480 × 480 over a field view of 240 mm.

### Magnetic resonance imaging data processing for cortical thickness measurements

The images were processed using the CIVET anatomical pipeline (version 2.1.0) (Zijdenbos et al., 2002). The native MRI images were registered to the Montreal Neurological Institute—152 templates by linear transformation (Collins et al., 1994) and corrected for intensity non-uniformities using the N3 algorithm (Sled et al., 1998). The registered and corrected images were divided into white matter, gray matter, cerebrospinal fluid, and background. The constrained Laplacian-based automated segmentation with proximity algorithm (Kim et al., 2005) automatically extracts the surfaces of the inner and outer cortices. The inner and outer surfaces had the same number of vertices, and there were close correspondences between the vertices of the inner and outer cortical surfaces. Cortical thickness, defined as the Euclidean distance between the linked vertices of the inner and outer surfaces (Lerch and Evans, 2005), was not calculated in Talairach spaces but in native brain spaces because of the limit of linear stereotaxic normalization. As expected, there was a significant positive correlation between cortical thickness and intracranial volume (ICV) in the native space (Im et al., 2008). Controlling for ICV, which reflects the brain size effect, is necessary to compare cortical thickness among participants. In a previous study (Im et al., 2008), the measurement of native space cortical thickness, followed by analyses that include brain size as a covariate, is an efficient method that explains the relationship between cortical thickness and brain size in depth. The ICV is defined as the total volume of gray matter, white matter, and cerebrospinal fluid. It was calculated by measuring the total volumes of the voxels within the brain mask, which were obtained *via* functional MRI of the brain software library bet algorithm (Smith, 2002). Cortical surface models were extracted from MRI volumes transformed into stereotaxic space, and cortical thickness was measured in the native space by applying an inverse transformation matrix to the cortical surfaces and reconstructing them in the native space (Im et al., 2006).

We applied surface-based two-dimensional registration with a sphere-to-sphere warping algorithm and spatially normalized the cortical thickness values to compare the thickness of corresponding regions among subjects. We used an improved surface registration algorithm and an unbiased iterative group template showing enhanced anatomic detail (Lyttelton et al., 2007) to transform the thickness information of the vertices into an unbiased iterative group template.

Surface-based diffusion smoothing with a full-width at half-maximum of 20 mm was used to blur each cortical thickness map, which increased the signal-to-noise ratio and statistical power (Chung et al., 2003; Im et al., 2006).

Thirteen patients with PPA were excluded due to errors in the analyses of cortical thickness, including CIVET pipeline errors (*n* = 6) and gray-white matter segmentation errors due to severe atrophy (*n* = 7). Therefore, a total of 96 patients with PPA were included in the main analyses.

### Magnetic resonance imaging data processing for cortical thickness measurement

As described in a previous study (Kang et al., 2019) to measure asymmetric degrees of neural substrates for language function tests, we acquired an asymmetric index (AI), which was calculated using the following formula: (R − L/R + L), where R indicates the number of vertices with significant correlations in the right hemisphere and L indicates the number of vertices with significant correlations in the left hemisphere. After acquiring the AI, we divided the extent of asymmetry into three groups according to the absolute AI value. When the absolute value of AI was ≤0.1, >0.1, and ≤0.5, or >0.5, we classified the cases as no hemispheric dominance, weak hemispheric dominance, or strong hemispheric dominance, respectively.

### Statistical analyses

We used analysis of variance and chi-square tests to compare the clinical demographic data and the results of the language function tests between the PPA subtype groups. SPSS version 25.0 (SPSS Inc., Chicago, IL, United States) was used to analyze the statistical data. Statistical significance was set at *p* < 0.05.

For cortical thickness analyses of MRI data from patients with PPA, we used a MATLAB-based toolbox (freely available online at the University of Chicago website: http://galton.uchicago.edu/faculty/InMemoriam/worsley/research/surfstat/). To identify the cortical atrophy pattern in the three PPA subtypes, we analyzed localized differences in cortical thickness between the NC and PPA subtype groups using a general linear model after controlling for age, sex, years of education, AQ, and ICV.

In order to investigate the correlation between each language function test score and cortical thickness in patients with PPA, we entered cortical thickness data into the dependent variable and language scores into the independent variable due to the high dimensionality of cortical thickness data (about 80,000 vertices). To check the consistency in the neural substrate depending on the ratio of PPA subtypes, we further performed a sensitivity analysis after excluding the 26 patients with nfvPPA to change the ratio of PPA subtypes (20 nfvPPA, 30 svPPA, and 20 lvPPA). Linear regression was performed after controlling for age, sex, years of education, and ICV as covariates. The statistical maps were thresholded using random field theory (RFT) at *p* < 0.05.

## Results

### Clinical characteristics of the patients

Table 1 shows the baseline demographics of the patients with PPA. The mean baseline age was 67.1 ± 8.9 years and 46 out of 96 (47.9%) were women. The mean years of education were 11.6 ± 4.4 and the mean AQ score was 65.9 ± 21.4. All patients were right-handed. In terms of each PPA subtype, patients with svPPA had higher years of education (13.8 ± 2.7 years) than those with nfvPPA (11.5 ± 4.8 years) and lvPPA (10.2 ± 4.4 years). There was no difference in age (*p* = 0.089), sex ratio (*p* = 0.920), disease duration (*p* = 0.558), AQ score (*p* = 0.853), and CDR (*p* = 0.950) between the three groups.

**TABLE 1:** Demographic variables and AQ of the patients with primary progressive aphasia.

	Total (*n* = 96)	nfvPPA (*n* = 46)	svPPA (*n* = 30)	lvPPA (*n* = 20)	*p*-value	NC (*n* = 308)
Age	67.1 ± 8.9	69.0 ± 9.2	66.8 ± 9.9	64.5 ± 7.0	0.089	69.5 ± 8.4*
Sex (M:W)	46:50	23:23	14:16	9:11	0.920	189:119*
Duration (m)	29.0 ± 16.9	29.2 ± 19.6	30.9 ± 15.0	25.7 ± 12.2	0.558	
Education (y)	11.6 ± 4.4	11.5 ± 4.8	13.8 ± 2.7	10.2 ± 4.4	0.019	11.9 ± 4.7
AQ	65.9 ± 21.4	65.5 ± 22.3	68.3 ± 21.3	65.0 ± 20.7	0.853	
CDR					0.794	
0/0.5	56 (58.3%)	26 (56.5%)	17 (56.7%)	13 (65.0%)		
1/2/3	40 (41.7%)	20 (43.5%)	13 (43.3%)	7 (35.0%)		

Values are presented as the mean ± standard deviation. The *p*-values were obtained using analysis of variance, chi-square tests between three PPA subtypes.

*Normal control had older age and higher men ratio than patients with PPA. AQ, aphasia quotient; CDR, clinical dementia rating; lvPPA, logopenic variant primary progressive aphasia; M, men; m, month; n, number of patients whose data were available for analysis; NC, normal control; nfvPPA, non-fluent variant primary progressive aphasia; svPPA, semantic variant primary progressive aphasia; W, women; y, year.

### Topography of cortical thinning in patients with subtypes of primary progressive aphasia

**FIGURE 1:**
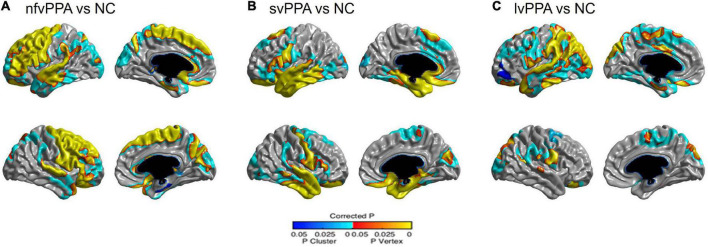
Statistical representation of cortical thickness in **(A)** nfvPPA compared to NC, **(B)** svPPA compared to NC, and **(C)** lvPPA compared to NC. nfvPPA, non-fluent variant primary progressive aphasia; svPPA, semantic variant primary progressive aphasia; lvPPA, logopenic variant primary progressive aphasia; NC, normal controls.

Figure 1 shows the topography of cortical thinning in patients with PPA subtypes. Compared to participants with NC, patients with nfvPPA mainly exhibited cortical thinning in the bilateral prefrontal (superior, middle, inferior, and medial frontal), left superior and middle temporal, right superior temporal, bilateral parietal, and right occipital regions. Patients with svPPA mainly exhibited cortical thinning in the bilateral temporal (superior, middle, inferior, and anterior temporal) and inferior frontal regions. Patients with lvPPA exhibited significant cortical thinning in the left superior/inferior/anterior temporal, superior/inferior parietal, and occipital regions and right inferior parietal regions.

### Correlation between language function tests and cortical thickness

**FIGURE 2:**
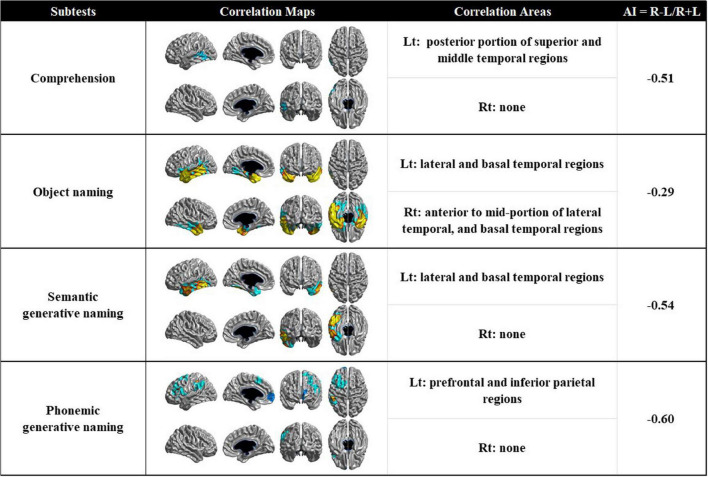
Correlation maps demonstrating the association between cortical thickness and language function tests in patients with PPA (AI > 0 means right-sided correlated areas > left-sided correlated areas, and vice versa for AI < 0). AI, asymmetric index; Rt, right; Lt, left.

The statistical map showed that lower cortical thickness in specific brain regions was associated with raw scores on language function tests, including comprehension, object naming, semantic generative naming, and phonemic generative naming, whereas the scores for language fluency and sentence repetition were not (Figure 2). Specifically, the score on the comprehension test was positively associated with cortical thickness in the left posterior portion of the superior and middle temporal regions. The score on the object naming test was positively associated with cortical thickness in the bilateral anterior to mid-portion of the lateral temporal and basal temporal regions. The score on the semantic generative naming test was positively associated with cortical thickness in the left anterior to mid-portion of the lateral and basal temporal regions. The score on the phonemic generative naming test was positively associated with cortical thickness in the left prefrontal and inferior parietal regions. After the threshold of the statistical map was changed from RFT correction at a *p*-value of 0.05 to an uncorrected *p*-value of 0.01, the score in sentence repetition was positively associated with the posterior portion of the left superior temporal, temporo-parietal junction, and inferior parietal regions (Supplementary Figure 1).

We explored the hemispheric dominance of neural substrates using the AI value. Neural substrates for comprehension, semantic generative naming, and phonemic generative naming have a strong left hemispheric dominance.

### Sensitivity analysis

Among 70 patients with PPA (20 nfvPPA, 30 svPPA, and 20 lvPPA), the neural substrate of object naming tests was similar to those in main results, while the correlations between the other language function tests (comprehension, semantic generative naming, and phonemic generative naming) and cortical thickness were not significant, because the sample size was small (Supplementary Figure 2). However, after the threshold of the statistical map was changed from RFT correction to an uncorrected *p*-value of 0.05, the patterns in neural substrates of semantic and phonemic generative naming test were similar to those in main results. The neural substrate of comprehension tests were bilateral inferior temporal and inferior parietal regions, which was partially consistent with those in main results.

## Discussion

In this study, we investigated the neural substrates of language function tests in a relatively large cohort of Korean patients with PPA. Our major findings are as follows: First, the three PPA subtypes showed distinct cortical atrophy patterns, which are consistent with previous studies from Western countries. Second, poor performance on each language function test was associated with lower cortical thickness in specific cortical regions. Specifically, object naming and semantic generative naming had similar neural substrates, whereas, different neural substrates were manifested in each of the generative naming tests: semantic and phonemic: the bilateral anterior to mid-portion of the lateral temporal and basal temporal regions in the object naming test, left anterior to mid-portion of the lateral and basal temporal regions in the semantic generative naming test, and left prefrontal and inferior parietal regions in the phonemic generative naming test. In particular, the neural substrates related to the semantic generative naming test in patients with PPA quite differ from previously reported results of patients with other causes of dementia. Taken together, our findings suggest that the neural substrates of language performance in Korean patients with PPA are specific to each language domain. Furthermore, a better understanding of the neural substrates specific to PPA might help clinicians understand the neural network of each language component and predict cortical atrophy patterns from the results of language function tests in Korean patients with PPA.

In the present study, the PPA subtypes showed distinct patterns of cortical atrophy. Specifically, we identified prefrontal and temporal atrophy in the nfvPPA, inferior frontal and anterior to mid-portion of temporal atrophy in the svPPA, and superior temporal and inferior parietal atrophy in the lvPPA. Our findings are consistent with those of previous studies conducted in Western countries (Sapolsky et al., 2010; Mesulam et al., 2014). However, there were several overlaps in atrophy across the PPA subtypes: left temporal atrophy in all three PPA subtypes and left inferior frontal atrophy in nfvPPA and svPPA. This finding may be due to higher proportion of middle-stage PPA in the present study compared to the previous studies (Sapolsky et al., 2010). Therefore, our findings suggest that the patterns of cortical atrophy in the three PPA subtypes lead to the development of corresponding clinical syndromes, regardless of differences in language.

Our major finding was that poor performance on each language function test was associated with lower cortical thickness in specific regions. Each naming test yielded different neural substrates. Specifically, poor performance in the object naming test was associated with lower cortical thickness in the bilateral (left predominant) anterior to the mid-portion of the lateral temporal and basal temporal regions. Our findings were consistent with those of previous studies investigating neural substrates for object naming in patients with PPA (Patterson et al., 2007; Hurley et al., 2012; Mesulam et al., 2013, 2019). The studies consistently suggested that anterior temporal atrophy was responsible for impaired object naming function observed in PPA patients. The basal temporal regions, which are one of the neural substrates of object naming in the present study, are reported to be vital to object naming. Previous MRI studies with patients with AD or SVCI revealed consistent findings that poor performance in BNT, which is a different evaluation tool for object naming, was associated with basal temporal regions (Ahn et al., 2011; Baldo et al., 2013; Kang et al., 2019). However, our study revealed that the mid-portion of temporal atrophy was associated with impaired object naming as well. According to the progression patterns of cortical atrophy in PPA, mid-portion temporal atrophy was accompanied by anterior temporal atrophy (Rogalski et al., 2011b); therefore, it might be reasonable to expect a result related to the mid-portion of temporal atrophy.

Unlike object naming tests, word fluency tasks rely on frontal executive control processes, such as initiation, self-monitoring, cognitive flexibility, and inhibition of dominant responses (Miyake and Friedman, 2012), as well as language-related functions, such as accessing semantic and lexical knowledge to identify related items (Henry and Crawford, 2004), lexical access ability (Shao et al., 2014), and language processing abilities (Whiteside et al., 2016). However, in the present study, poor performance in the semantic generative naming test was mainly associated with lower cortical thickness in the left anterior to mid-portion of the lateral and basal temporal regions. Our findings are inconsistent with those of previous studies on other causes of dementia (Kang et al., 2019, 2021). The previous studies showed that the neural substrates for semantic generative naming turned out to be frontal regions as well as anterior and inferior temporal regions in AD patients,(Kang et al., 2019) and only superior and inferior frontal regions (but not temporal regions) in SVCI patients (Kang et al., 2019, 2021).

It is important to know the reason for the different patterns of the neural substrates related to the semantic generative naming test between our results and previous reported results of patients with other causes of dementia because it might provide a better understanding of the different pathomechanisms for semantic generative naming dysfunction among these patients. The ability to access semantic and lexical knowledge is required to perform semantic word fluency tasks (Gordon et al., 2018). One of the PPA subtypes (i.e., svPPA) manifests with severe deficits in semantic processing associated with temporal regions (Wilson et al., 2014). Severe semantic processing dysfunction in patients with PPA causes them to struggle to come up with a word within a category, which may leave no room for frontal executive functions to play a role in performing the task (Riello et al., 2021). In contrast, the previous results shown in patients with AD may reflect the fact that both temporal and frontal functions affect their performance on this task (Ahn et al., 2011; Kang et al., 2019). In addition, in patients with SVCI, because their semantic system is somewhat intact, frontal dysfunction may be the main function influencing their performance on this task (Kang et al., 2021). In fact, our findings were consistent with those of a recent study based on patients with PPA, which showed that poor performance in the semantic generative naming test was associated with lower cortical thickness in left lateral temporal region (Riello et al., 2021).

Moreover, we found that poor performance on the phonemic generative naming test was associated with lower cortical thickness in the left prefrontal and inferior parietal regions. These findings are in line with those of previous studies in patients with AD and SVCI (Keilp et al., 1999; Kitabayashi et al., 2001; Kang et al., 2021). A recent study based on PPA patients revealed that poor performances in the phonemic generative naming test were associated with prefrontal atrophy (Riello et al., 2021). Phonemic word fluency tasks may require access to a certain phoneme and active frontal executive functions that include self-monitoring, set shifting, and applying different strategies to come up with words that satisfy the target phoneme (Dilkina et al., 2010; Biesbroek et al., 2021). The inferior parietal region has previously been reported to be related to phonologic errors, which might indicate partial difficulty in appropriate phoneme access (Petroi et al., 2020). lvPPA manifests difficulties in phonological processing, involving atrophy in the inferior parietal region. Additionally, nfvPPA is characterized by frontal executive dysfunction and atrophy of the frontal region. Therefore, our neuroimaging findings of the frontal and inferior parietal atrophies related to the phonemic generative naming test may reflect the phoneme access functions and frontal executive functions required to perform this task. Meta-analysis also revealed that the phonemic generative naming test mainly relies on frontal executive function, which supports our finding (Henry and Crawford, 2004).

We found that impairment of overall comprehension was associated with lower cortical thickness in the left posterior portion of the superior and middle temporal regions. Meta-analyses investigating the neural substrates of comprehension have reported that the temporal lobe plays an important role in various comprehension tasks (Ferstl et al., 2008; Binder et al., 2009). Recent studies with PPA suggested that the anterior portion of the temporal region was associated with comprehension (Rogalski et al., 2011a; Mesulam et al., 2015, 2019), which was different from classic aphasiology. Prior anatomical studies of stroke lesions have shown that Wernicke’s area plays an important role in comprehension (Robson et al., 2013; Hillis et al., 2017). Given that the neural substrates in our study included Wernicke’s area, our results support the findings of stroke studies. An explanation for these discrepant results between our study and previous studies using PPA patients is the difference in the components of comprehension. Previous studies have explored the neural substrates of only word comprehension rather than overall comprehension. However, comprehension tests on the K-WAB include word comprehension, sentence comprehension, and command comprehension.

Poor sentence repetition performance was not associated with lower cortical thickness in any of the regions. However, before RFT correction, there was a trend of positive correlation between the score on sentence repetition and cortical thickness in the posterior portion of the superior temporal, temporo-parietal junction, and inferior parietal regions, which was significant at an uncorrected *p*-value of 0.01. These regions include Wernicke’s area associated with comprehension rather than repetition by classic aphasiology. However, these regions were consistent with known neural substrates of repetition in patients with PPA (Amici et al., 2007; Mesulam et al., 2019; Forkel et al., 2020). Furthermore, previous studies have revealed that the temporoparietal junction is vital for the integrity of phonological encoding and auditory working memory necessary for repetition (Gorno-Tempini et al., 2008; Binder, 2015, 2017).

In the present study, no specific cortical atrophy regions were identified in the language fluency task, although previous studies with English-speaking patients with PPA showed that the inferior and middle frontal regions were mainly correlated with language fluency (Sapolsky et al., 2010; Rogalski et al., 2011a). We have used the 10-point fluency scores (fluency, grammatical competence, and paraphasia in K-WAB) to see the associated cortical areas with language fluency, which showed null results. Since the Korean language is somewhat less strict in grammar compared to English, there is no adequate evaluation tool that can be used to detect grammatical processing in Korean. Further research using detailed utterance analysis based on the transcription of spontaneous speech performances may enable us to detect areas specific to fluency characteristics in Korean.

According to our expectations, poor performance in most language function tests was more likely to be associated with lower cortical thickness in the left hemisphere, and left hemispheric dominance were strong. The neural substrates of overall comprehension, sentence repetition, semantic generative naming, and phonemic generative naming were strongly lateralized in the left hemisphere. This may also be explained by the left-dominant cerebral atrophy pattern in patients with PPA. Contrary to expectations, however, the neural substrate of object naming had weak left hemispheric dominance. The absence of lateralization in object naming has been reported previously (Teipel et al., 2006; Apostolova et al., 2008). Another study showed that right anterior temporal area was responsible for making semantic decision, although the significance of the right hemisphere for naming is not fully understood. Some researchers have suggested that the neural substrate of naming is primarily lateralized to the left hemisphere and becomes bilateral during the aging process (Cabeza, 2002). Actually, one study comparing naming performance between younger and older adults showed that older participants were more likely to have bilateral activation than younger participants (Wierenga et al., 2008). The other MRI study observed a positive correlation between naming ability and bilateral gray matter volume in older participants (Obler et al., 2010). Increasing involvement of the right hemisphere might be explained by the compensation for brain atrophy with aging. Given that the patients in our study were elderly, our findings are in line with those of previous studies.

In the present study, we investigated the neural substrates of language function tests in a relatively large sample of Korean patients with PPA. However, this study has several limitations. First, although we showed that specific cortical regions were associated with poor performance on language function tests, it is doubtful that all cortical regions that we found represent test-specific cortical areas. Rather, some neural substrates for each language function test may be relevant to basic language or cognitive processes shared by a variety of cognitive functions. Second, we used the measure of cortical thickness representing the gray matter structure to explore neural substrates. Thus, we did not include changes in white matter structure. However, this argument has been mitigated to some degree by the fact that PPA is a neurodegenerative disease that primarily affects the cerebral cortex. Third, we did not assess the cerebral dysfunction which precedes the cortical atrophy. Finally, we could not perform a direct comparison of neural substrates between patients with Korean-speaking PPA and those with AD or between those with Korean-speaking PPA and those with English-speaking PPA. Nevertheless, our study is noteworthy because it was based on Korean patients who had a different language and culture from English-speaking PPA patients. Furthermore, our findings may help clinicians diagnose PPA and predict disease progression through a better understanding of the neural substrates of language impairment in patients with PPA.

In conclusion, Korean patients with PPA showed that poor performance in each language function test was associated with atrophy in specific cortical regions. In particular, the neural substrates of the semantic generative naming test in patients with PPA quite differ from those in patients with other causes of dementia. This might provide a better understanding of the different pathomechanisms of language impairment among these patients.

## Data availability statement

The raw data supporting the conclusions of this article will be made available by the authors, without undue reservation.

## Ethics statement

This study was approved by the Institutional Review Board of the Samsung Medical Center. Written informed consent was obtained from all the patients or their caregivers. The patients/participants provided their written informed consent to participate in this study.

## Author contributions

SK, MS, and SS: concept and design. JS, JY, H-RK, HJ, HK, S-BK, DN, MS, and SS: acquisition of data. SK, YP, MS, and SS: analysis or interpretation of data. SK: drafting of the manuscript. SK, S-BK, MS, and SS: intellectual content. SK and YP: statistical analysis. MS and SS: administrative, technical, or material support and supervision. All authors contributed to the article and approved the submitted version.
