# Supplementary material for: Cortical neuroanatomical changes related to specific language impairments in primary progressive aphasia
Source: Front Aging Neurosci. 2022 Aug 25;14:878758. doi: 10.3389/fnagi.2022.878758 (PMC9452784; doi:10.3389/fnagi.2022.878758)
Supplement: Supplementary file 1 [file Data_Sheet_1.docx]

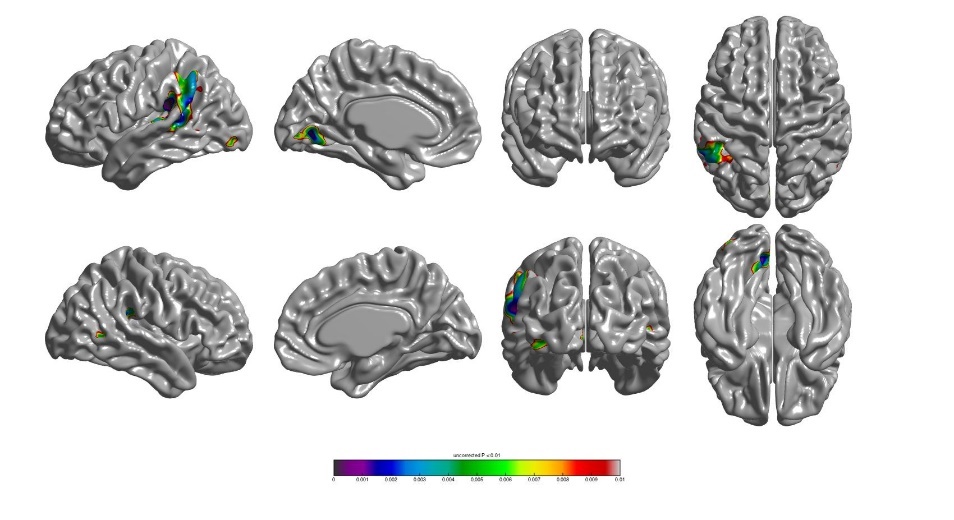


**Supplementary Figure 1.** Correlation maps demonstrating the association between cortical thickness and sentence repetition score in PPA patients with an uncorrected *p*-value of 0.01


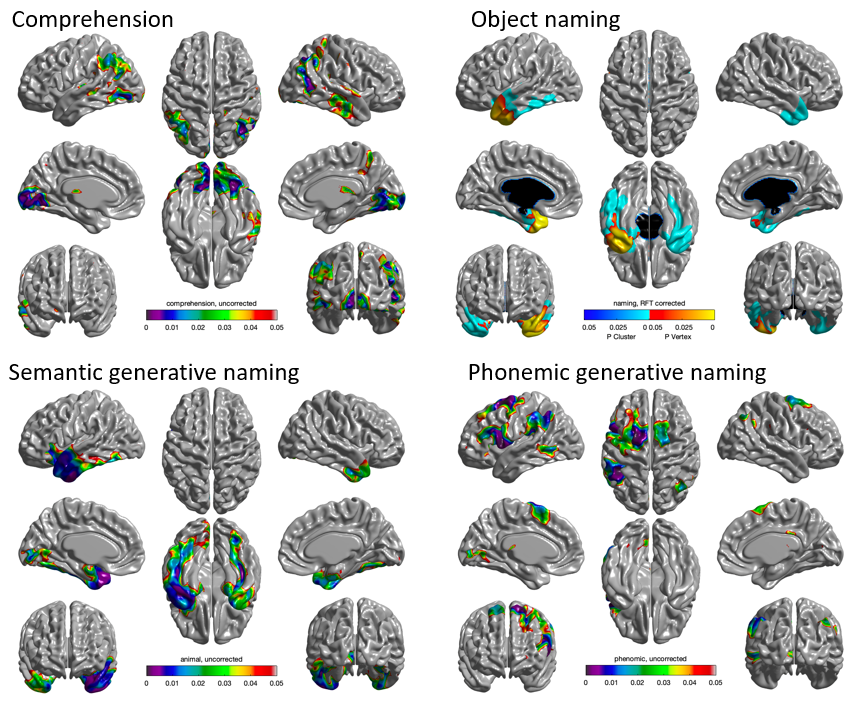


**Supplementary Figure 2.** Correlation maps demonstrating the association between cortical thickness in 70 patients with PPA (20 nfvPPA, 30 svPPA, and 20 lvPPA). The statistical maps were thresholded using random field theory (RFT) at *p* < 0.05 in object naming test and uncorrected *p*-value of 0.01 in comprehension, semantic generative naming, and phonemic generative naming tests.
